# Widespread position-specific conservation of synonymous rare codons within coding sequences

**DOI:** 10.1371/journal.pcbi.1005531

**Published:** 2017-05-05

**Authors:** Julie L. Chaney, Aaron Steele, Rory Carmichael, Anabel Rodriguez, Alicia T. Specht, Kim Ngo, Jun Li, Scott Emrich, Patricia L. Clark

**Affiliations:** 1 Department of Chemistry & Biochemistry, University of Notre Dame, Notre Dame, Indiana, United States of America; 2 Department of Computer Science & Engineering, University of Notre Dame, Notre Dame, Indiana, United States of America; 3 Department of Applied and Computational Mathematics & Statistics, University of Notre Dame, Notre Dame, Indiana, United States of America; 4 Department of Chemical & Biomolecular Engineering, University of Notre Dame, Notre Dame, Indiana, United States of America; University of Texas at Austin, UNITED STATES

## Abstract

Synonymous rare codons are considered to be sub-optimal for gene expression because they are translated more slowly than common codons. Yet surprisingly, many protein coding sequences include large clusters of synonymous rare codons. Rare codons at the 5’ terminus of coding sequences have been shown to increase translational efficiency. Although a general functional role for synonymous rare codons farther within coding sequences has not yet been established, several recent reports have identified rare-to-common synonymous codon substitutions that impair folding of the encoded protein. Here we test the hypothesis that although the usage frequencies of synonymous codons change from organism to organism, codon rarity will be conserved at specific positions in a set of homologous coding sequences, for example to tune translation rate without altering a protein sequence. Such conservation of rarity–rather than specific codon identity–could coordinate co-translational folding of the encoded protein. We demonstrate that many rare codon cluster positions are indeed conserved within homologous coding sequences across diverse eukaryotic, bacterial, and archaeal species, suggesting they result from positive selection and have a functional role. Most conserved rare codon clusters occur within rather than between conserved protein domains, challenging the view that their primary function is to facilitate co-translational folding after synthesis of an autonomous structural unit. Instead, many conserved rare codon clusters separate smaller protein structural motifs within structural domains. These smaller motifs typically fold faster than an entire domain, on a time scale more consistent with translation rate modulation by synonymous codon usage. While proteins with conserved rare codon clusters are structurally and functionally diverse, they are enriched in functions associated with organism growth and development, suggesting an important role for synonymous codon usage in organism physiology. The identification of conserved rare codon clusters advances our understanding of distinct, functional roles for otherwise synonymous codons and enables experimental testing of the impact of synonymous codon usage on the production of functional proteins.

## Author summary

Proteins are long linear polymers that must fold into complex three-dimensional shapes in order to carry out their cellular functions. Every protein is synthesized by the ribosome, which decodes each trinucleotide codon in an mRNA coding sequence in order to select the amino acid residue that will occupy each position in the protein sequence. Most amino acids can be encoded by more than one codon, but these synonymous codons are not used with equal frequency. Rare codons are associated with generally slower rates for protein synthesis, and for this reason have traditionally been considered mildly deleterious for efficient protein production. However, because synonymous codon substitutions do not change the sequence of the encoded protein, the majority view is that they merely reflect genomic ‘background noise’. To the contrary, here we show that the positions of many synonymous rare codons are conserved in mRNA sequences that encode structurally similar proteins from a diverse range of organisms. These results suggest that rare codons have a functional role related to the production of functional proteins, potentially to regulate the rate of protein synthesis and the earliest steps of protein folding, while synthesis is still underway.

## Introduction

Most amino acids are encoded by multiple codons, but these synonymous codons are not used with equal frequency. Rare codons generally correlate with lower levels of cognate tRNA, or weaker codon:anticodon interactions [*1,2*]. As a result, rare codons are generally associated with slower translation rates and are typically considered deleterious, due to their negative impact on high level gene expression [*3*] and sometimes lower translational accuracy [*4*]. The conventional view holds that selection favors common codons, which are considered translationally optimal, but a low level of rare codons is incorporated due to random mutational drift and weak selection [*5*]. However, the distribution of rare codons is non-random: clusters of synonymous rare codons are widespread in the coding sequences of most prokaryotic and eukaryotic species [*6,7*]. Clustering would be expected to exacerbate negative effects of rare codons. This suggests that the distribution of rare and common codons may be shaped by selection and plays a functional role in protein production.

Supporting a functional role for synonymous rare codons, altering synonymous codon usage has been shown to adversely affect the expression level [*8,9*], solubility [*10*] and co-translational modifications [*11*] of encoded proteins, and is hypothesized to regulate targeting of exported proteins [*12,13*]. Codon usage can also affect translational efficiency indirectly via mRNA structure effects at the 5’ end of coding sequences [*14–18*]. Within coding sequences, an intriguing hypothesis suggests that rare codons may slow translation rate to coordinate proper co-translational folding of the nascent polypeptide chain [*19–23*], potentially to simplify the folding energy landscape for multi-domain proteins [*24,25*]. Such effects have been observed for *in vitro* translation reactions of some proteins [*22,26*].

While previous studies have suggested that synonymous codon usage is functionally important for some proteins, it is not yet clear in which cases codon usage results from selection versus random drift. Efforts in this direction have been stymied in part because many past analyses of synonymous codon usage neglected to account for specific known biases in synonymous codon selection, including the percent GC content at the third nucleotide position of a codon [*27*], codon pair bias [*28*], low sequence divergence between recently duplicated genes (paralogs), and potentially other unknown sources of synonymous codon usage bias. Moreover, altering synonymous codon usage can affect gene expression in diverse ways [*29*]. In addition to the effects described above, synonymous mutations can also affect translational accuracy [*4,30,31*], splicing efficiency [*32,33*], and introduce undesirable nucleotide motifs such as internal Shine Dalgarno sites [*34*]. Some large clusters of synonymous rare codons have no measurable effect on protein folding [*6*]. In addition, even the rarest codons still encode ≥1% occurrences of an amino acid, challenging the identification of statistically significant usage patterns for functionally important rare codons against the background of neutral drift.

We hypothesized that synonymous rare codons that are important for co-translational protein folding might (*i*) occur in clusters [*6*], in order to produce larger translation rate changes than a single codon, and (*ii*) occur at similar positions amongst homologous proteins across the tree of life, as homologous proteins often have similar three dimensional structures [*35*]. Under this hypothesis, evolution would select for codon rarity at a particular position in an alignment of mRNA sequences without necessarily conserving a specific DNA or protein sequence. To test whether synonymous rare codon clusters are conserved during evolution, we developed a rigorous set of criteria, including an appropriate null model and statistical tests, to analyze codon usage in all open reading frames of 76 diverse, fully sequenced genomes. This analysis revealed a widespread conservation of synonymous rare codon clusters, particularly amongst water-soluble proteins, across diverse species. Most conserved rare codon clusters were found within conserved protein structural domains, rather than between domains. These results indicate that synonymous rare codons are frequently subject to positive selection, and have functional importance across the tree of life.

## Results

### Synonymous rare codon clusters are conserved among families of homologous genes

**Fig 1:**
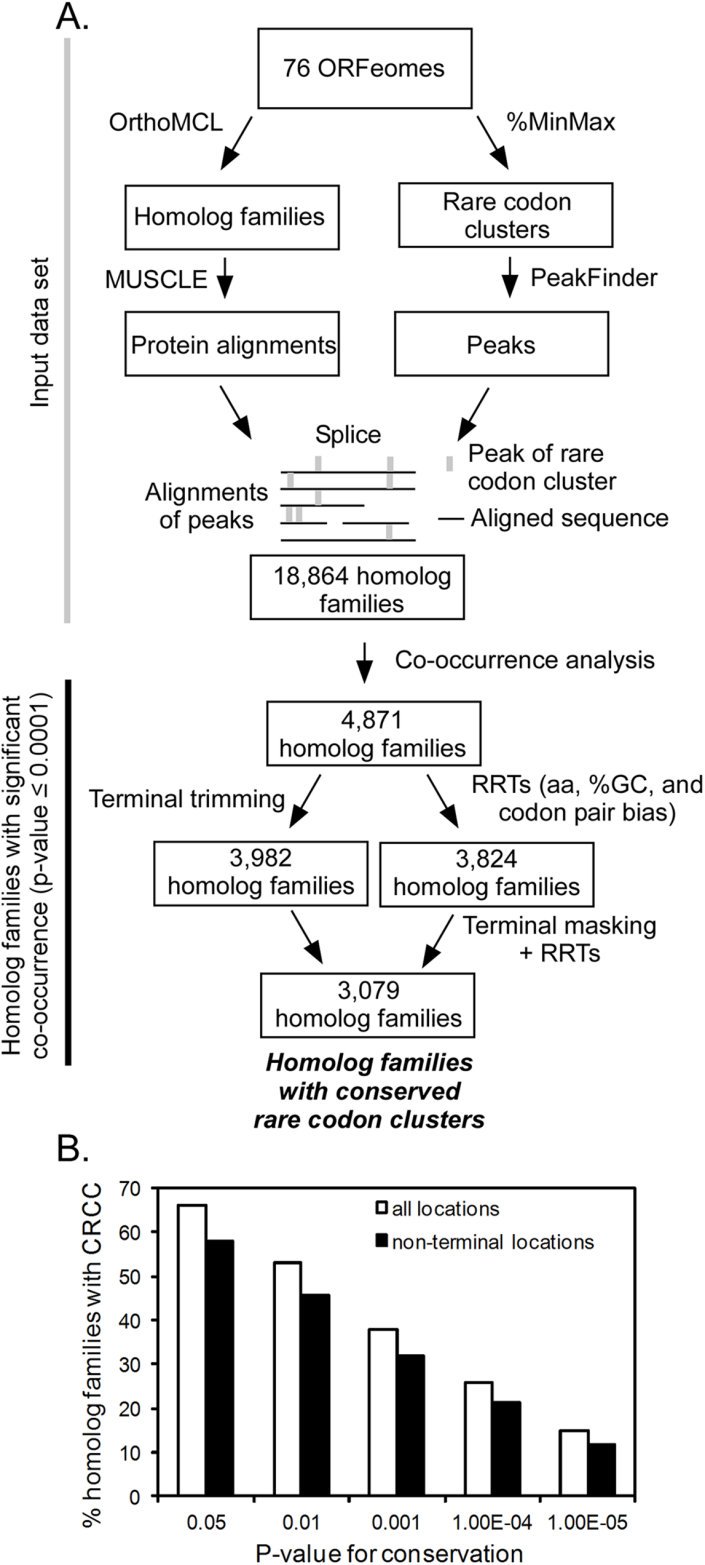
Identification and analysis of conserved rare codon clusters (CRCCs). (**A**) Overview of the analysis pipeline. From the ORFeomes of 76 species, homolog families were assigned using OrthoMCL [41]. Protein sequences within each family were aligned using MUSCLE [42]. ORFeome coding sequences were analyzed to locate rare codon clusters, and peak locations were spliced into alignments. These alignments of peaks were used as input for co-occurrence analysis. To avoid false positives due to closely related paralogs, homolog families were filtered to contain a maximum of one sequence from each species. Co-occurrence analysis determines if rare codon clusters in the resulting homolog families align more than expected by random chance. Terminal trimming removed 5’ and 3’ sequence termini, to identify co-occurrence of non-terminal rare codon clusters. Random reverse translations (RRTs) control for rare codon co-occurrence for reasons unrelated to codon rarity (see text for details). (**B**) Conservation of rare codons within homolog families is widespread. White bars indicate homolog families with conservation of rare codons at any position; black bars indicate homolog families with significant co-occurrence in regions other than the 5’ and 3’ termini.

A complete set of all annotated protein coding sequences (an ORFeome) was collected for each of 76 diverse eukaryotic, archaeal, and bacterial species with fully sequenced genomes (see Methods and **S1–S3 Tables**). Species were selected to span as much of the tree of life as possible (**S1 Fig**) in order to keep DNA identity low, as species with high DNA identity may have diverged too recently for synonymous codon conservation to be reliably detected. Protein sequences from these 76 ORFeomes were assigned to homolog families, and the sequences within each family were aligned (**Fig 1A**). To reduce potential false-positive results arising from recent gene duplications (paralogs), homolog families were trimmed to include only one sequence from each organism (see Methods).

The conserved codon usage patterns we sought to identify in these homolog families are those that do not alter the encoded amino acid sequence, as amino acid sequence changes can alter protein function, binding and/or stability. For this reason, we used the %MinMax algorithm [*6*] to analyze position-specific synonymous codon usage in each coding sequence. This algorithm compares the codon usage of the actual mRNA sequence to that of theoretical sequences encoding the same amino acid sequence using the most rare and common codons for each amino acid (see Methods), returning a value that reflects the relative rareness of the codons used to encode a specific amino acid sequence. A codon is defined as rare if its usage frequency within an ORFeome is less than the average usage frequency for codons within the same synonymous set [*6*]. In contrast, other codon usage calculators compare the absolute rarity of one codon versus all other 61 sense codons, which can reflect amino acid rarity due to unavoidable functional constraints on the amino acid sequence [*36*]. It has previously been shown that relative codon rarity is highly correlated with local translation rate [*37–39*] and changes to it can alter co-translational folding [*10,20,21,23*]. Changes to relative codon rarity correlate with changes in co-translational folding equally well as translation adaptive index (tAI) [*16*] (**S2 Fig**), but does not require fitting to an adjustable parameter.

Locations of synonymous rare codon clusters within the aligned coding sequences were determined, and this data was used as the input for conservation statistical analysis, which included three steps (**Fig 1A**). The initial step tested whether rare codon clusters in homolog families co-occur (align) more often than expected by random chance across the entire dataset. We counted the total number of rare codon cluster peaks that fall within a distance of +/- 2 positions across each homolog family, and compared this number of co-occurring rare codon clusters with the number from a null model where rare codon clusters were distributed randomly across coding sequences. We generated the null distribution by randomly shifting the protein sequence from each organism without distorting the positional relationships of codon usage (see Supplementary Information for a detailed description). This test returned a p-value <1x10^-300^, providing strong evidence that rare codon clusters as a whole tend to occur at the same positions across organisms.

The broad analysis described above determined that rare codon clusters in general show a non-random distribution, with 26% of homolog families showing significant rare codon co-occurrence (p-value < 1x10^-4^). However, it is possible that rare codon clusters might occur at the same positions in homologs for reasons unrelated to codon rarity, including amino acid bias, selection for %GC content, known codon pair biases [*39,40*] or other, unknown factors. Hence in the second step of the statistical analysis the homolog families with significant rare codon co-occurrence were filtered to remove families where co-occurrence did not differ significantly from co-occurrence found in random reverse translations (RRTs), a Monte Carlo simulation method we developed to control for rare codons that co-occur for reasons other than rarity (see Methods). Each RRT randomly generated an alternative mRNA sequence to encode an analyzed coding sequence without altering its amino acid sequence, based on the underlying codon usage frequencies of the host organism [*6*]. Crucially, these RRTs replicated the %GC content of each coding sequence and the codon pair biases of the species genome of origin (see Methods). This simulation generated a null model where co-occurrence of rare codons for reasons other than rarity could be detected using the same bioinformatics framework. This important null model control eliminated 1,047 homolog families; however, a substantial fraction (3,824; or 79%) of homolog families still showed significant (p-value ≤ 1x10^-4^) co-occurrence of rare codon clusters after adjusting for these effects (**Fig 1A**), indicating that these homolog families contain regions of conserved codon rarity.

In the third step of the statistical analysis, we filtered the dataset to determine what fraction of rare codon conservation arises due to previously observed conservation of rare codons at coding sequence termini in many species [*14–16,43*]. Homolog family alignments were trimmed to remove the first and last 50 codons and re-analyzed for co-occurrence (see Methods). The majority (3079, or 81%) of homolog families with significant conservation still showed significant conservation after this terminal trimming (**Fig 1B**), indicating widespread conservation of rare codon clusters within the interior of coding sequences.

### Conserved rare codon positions correlate with protein structure and function

**Fig 2:**
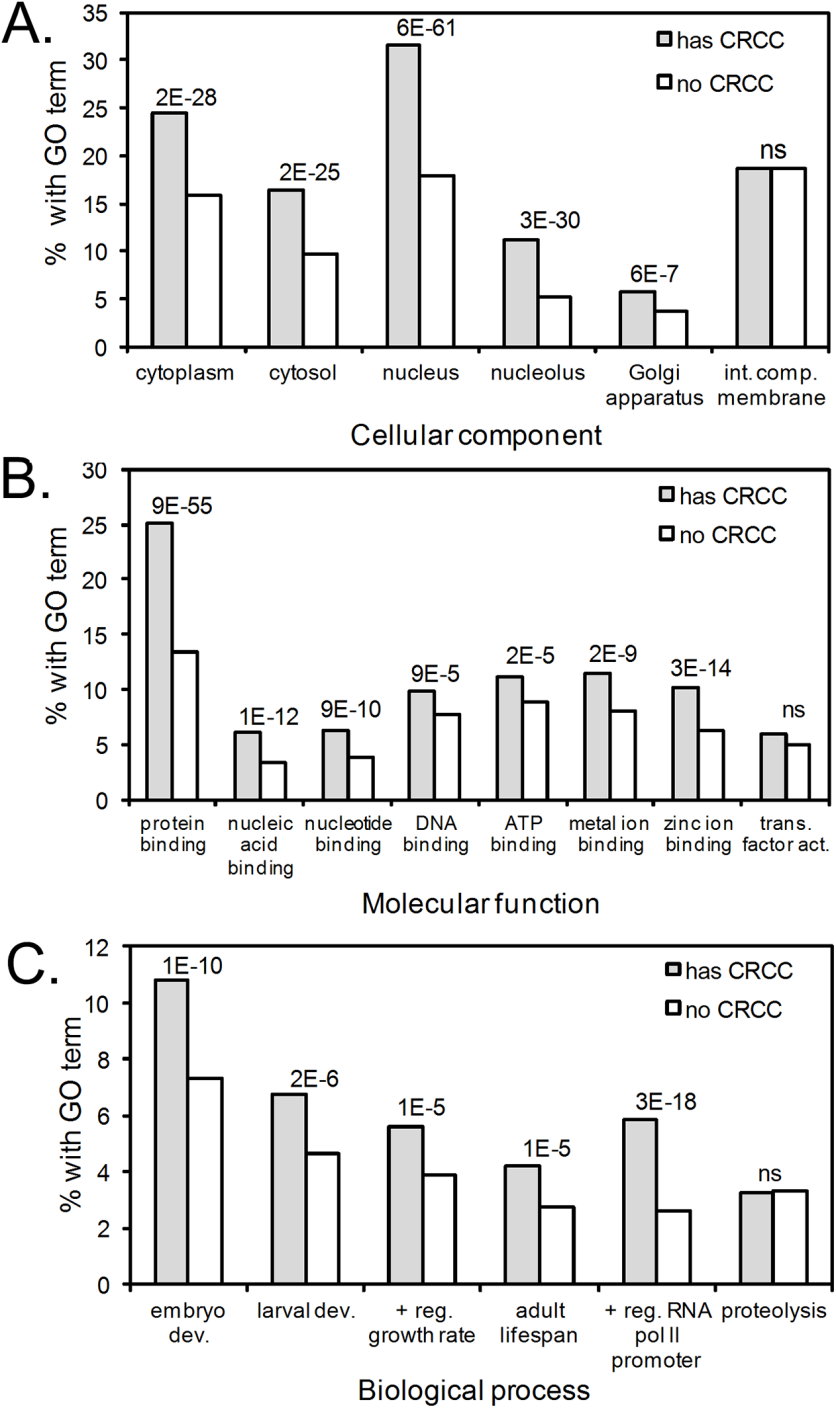
Homolog families with CRCCs are significantly enriched (p-value ≤ 0.0001) in certain gene ontology (GO) terms, including (**A**) cytosolic and nuclear localized proteins, (**B**) functions associated with binding, and (**C**) processes associated with growth and development. GO categories analyzed were the 10 most common GO terms in each GO term class (cellular component, molecular function, and biological process). The y-axis indicates the percentage of homolog families (with or without CRCC) that were assigned a particular GO term. Because the same homolog family may be assigned more than one GO term, bars will not necessarily sum to 100%. The right-most bars in each panel are included as examples of GO terms not significantly enriched in homolog families with CRCCs. No GO terms were significantly under-enriched in homolog families with CRCCs.

The conservation of rare codon clusters within homologous coding sequences implies that these synonymous codons are functionally important for protein biogenesis. To identify broad trends associated with conserved rare codon clusters (CRCCs), we first tested whether certain Gene Ontology (GO) categories are enriched and/or under-enriched among homolog families with CRCCs. We found significantly more CRCCs than expected in genes encoding water-soluble proteins that fold in the cytosol (i.e., cytosolic and nuclear proteins), proteins with functions associated with binding, and proteins that participate in processes associated with growth and development (**Fig 2**). The enrichment of water-soluble proteins may reflect differences in domain structure between water-soluble proteins and those that are membrane-bound. Several of the enriched protein functions, including DNA binding and promoter regulation, are associated with the enrichment in nuclear localization.

**Fig 3:**
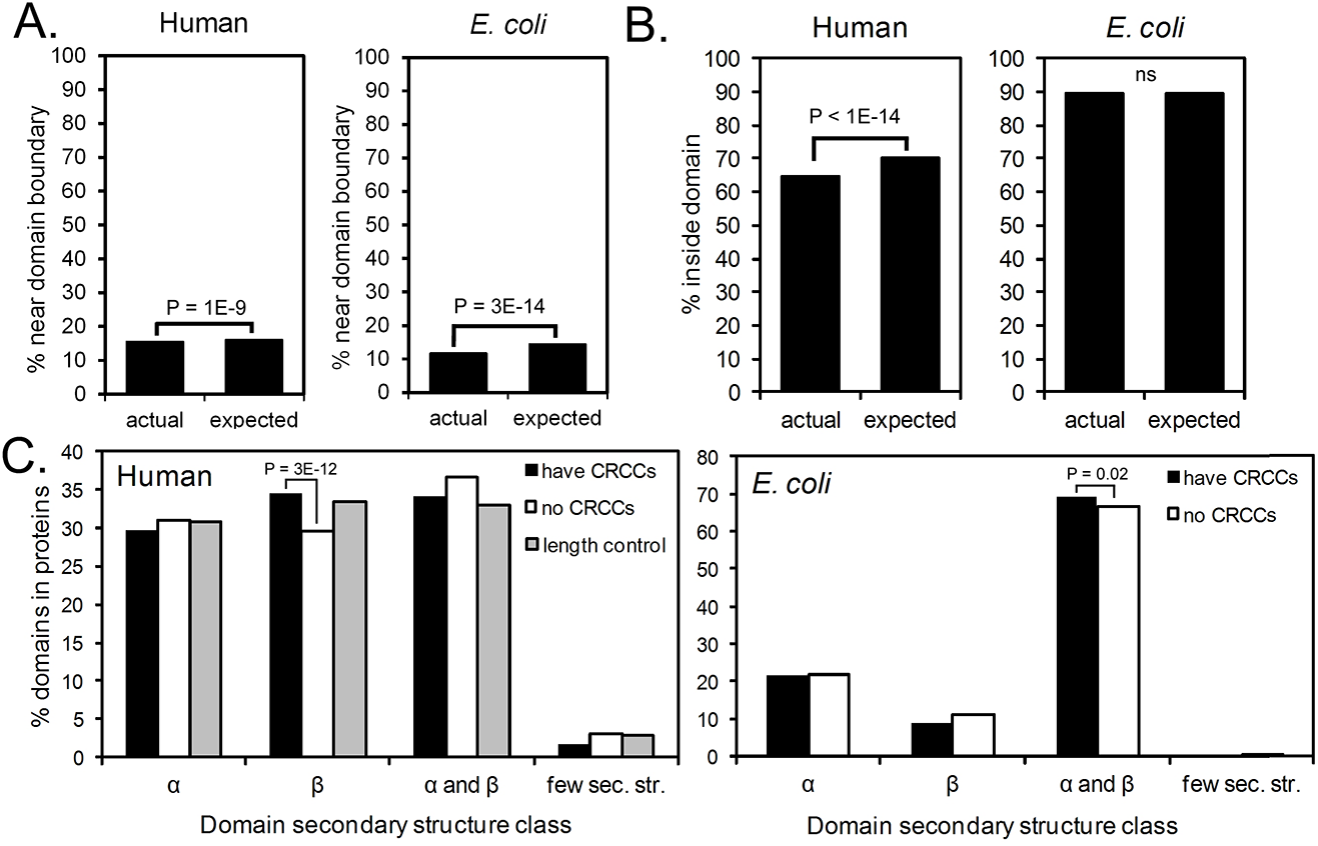
Positions of CRCCs relative to protein domains. (**A**) Comparison of the actual and expected frequency of CRCCs near (within +/- 20 codon positions) of domain boundaries. Expected frequency is based on random distribution, and p-values are calculated using a binomial test. (**B**) The majority of CRCCs in human and E. coli are found within mRNA regions encoding conserved structural domains. (**C**) Secondary structure classes of CATH domains in human and E. coli ORFeomes. CATH domains annotated by Gene3D were divided by secondary structure class. Graphs indicate the percent of domains that belong to each secondary structure class, for proteins in homolog families with (black) or without (white) conserved rare codon clusters (CRCC) (co-occurrence p-value ≤ 1E-4 after termini and RRT screening). Although human proteins from homolog families with CRCCs are significantly over-enriched in β-sheet-rich domains, this enrichment is most likely due to the longer average length of proteins with CRCCs, as similar results were seen for a control set of proteins with similar length but no CRCCs (gray).

**Fig 4:**
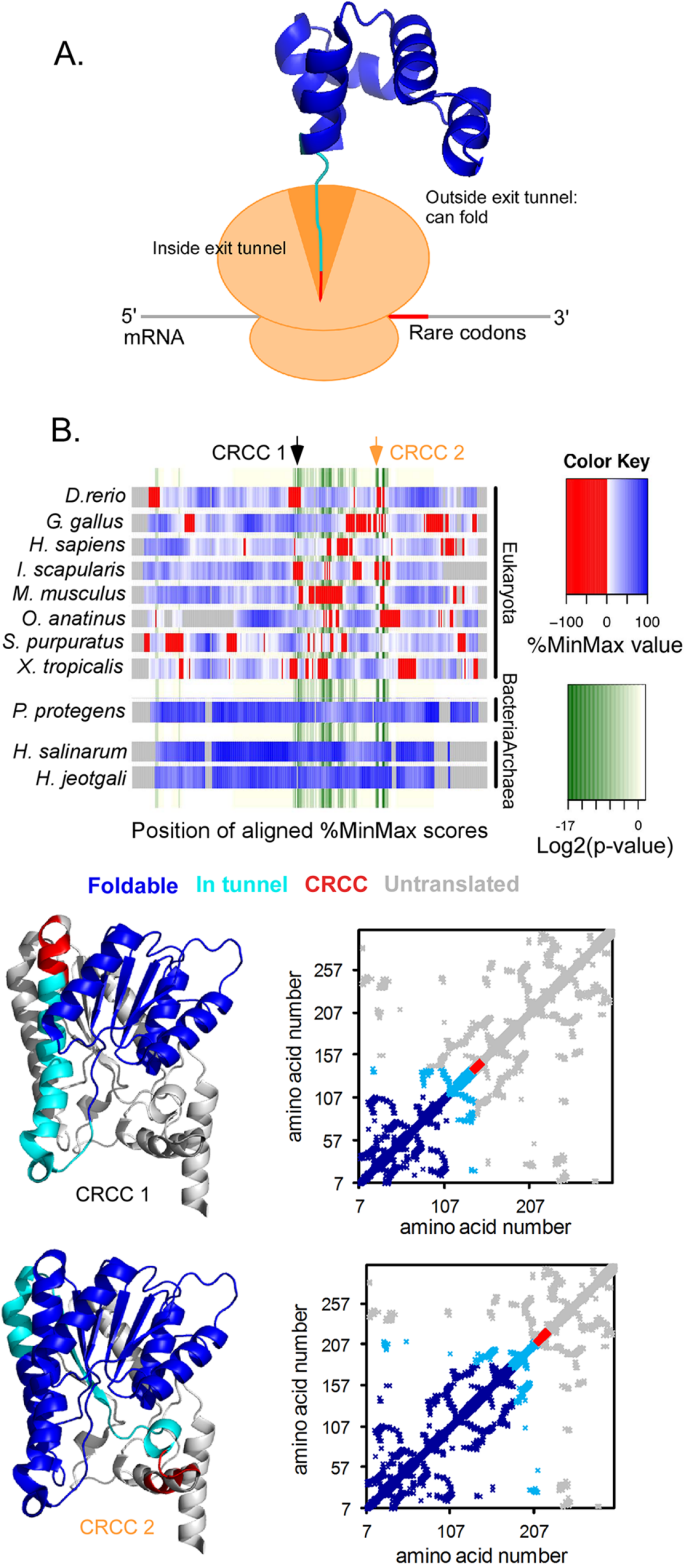
Connections between CRCC locations and co-translational folding units. (**A**) Schematic depicting the relative locations of a CRCC (red) to the portions of the nascent chain that have passed through the constriction of the ribosome exit tunnel (dark blue), versus the portion that remains within the tunnel (cyan). Ribosome is shown in orange. (**B**) A homolog family (peroxisomal trans 2-enoyl CoA reductase) with conserved rare codons. The heatmap represents the multiple sequence alignment of homologous protein sequences and indicates the location of rare codons within the alignment (clusters of rare codons are shown in red, regions with more common codons in blue). P-values for co-occurrence of rare codons in the sequence alignment are shown in a separate color scale (minimum p-value = 5E-6, indicated by dark green). The structure of the human homolog is shown (PDBID 1YXM). The structure is color-coded to indicate regions of the structure that would be outside the ribosome exit tunnel and able to fold at two of the possible rare codon-induced translational pauses: when the CRCC (red) is being translated, the N-terminal region of the protein (dark blue) would be able to fold. Locations of these CRCCs are indicated on the alignment by arrows. The contact maps indicate amino acids that are in contact (distance ≤ 6 angstroms) in the three-dimensional structure.

If CRCCs function to modulate co-translational folding of the encoded protein, we hypothesized that their positions might correlate with the locations of conserved structural features, particularly domain boundaries. Previous investigations of correlations between rare codons and domain boundaries have arrived at conflicting conclusions (e.g. [*24,44*]), perhaps because the analyses used small sets of proteins with solved structures. To broadly test whether CRCCs are enriched at or near the boundaries of protein structural domains, the locations of CRCCs were compared to the locations of annotated SCOP [*45*] and CATH [*46*] domains in proteins with PDB structures and domains predicted from gene sequences [*47*]. Surprisingly, this analysis revealed that CRCCs are significantly (p = 1E-9 for human, p = 3E-14 for *E*. *coli*) under-enriched near domain boundaries (**Fig 3A**). Hence the major function of CRCCs does not appear to be to separate the co-translational folding of entire domains. Instead, CRCCs often occurred at positions where a translational pause would expose a smaller structural sub-domain outside of the ribosome exit tunnel (**Fig 4, S4 Fig**). Crucially, small structural motifs such as these often fold much faster than an entire domain [*48–52*], and hence might be more sensitive to the small differences in the rate of appearance of the nascent chain achievable via synonymous codon selection.

It has been hypothesized that rare codons are enriched in unstructured regions of proteins due to reduced selection for translational accuracy in these regions [*53*]. Such an effect could potentially cause false positives in a study of rare codon conservation, if unstructured, poorly conserved regions in the homologs aligned. To avoid this issue, we focused on alignment regions with high amino acid conservation across homolog families; alignment columns containing gaps in any species were removed from consideration. To determine the fraction of CRCCs that occur in structured regions with conserved amino acid content, we compared to frequency of CRCCs inside and outside conserved domains (**Fig 3B**). The majority occurred inside conserved domains, suggesting that these rare codons are in fact conserved and do not result from neutral drift in regions where amino acid content is non-critical. However, although most CRCCs occurred inside conserved domains, a subset of CRCCs did show a small but statistically significant enrichment outside known conserved domains. This result highlights the complexity and challenges of a truly comprehensive analysis of synonymous codon usage. Going forward, novel computational methods will be required to distinguish between CRCCs with different roles.

### Connections between CRCCs and protein length and secondary structure

Coding sequences with CRCCs have higher average length than sequences without CRCCs (**S3 Fig**). This result is expected, as a longer length gives more opportunities for a CRCC to occur. Longer sequences do not, however, have a higher density of CRCCs per unit length than shorter sequences. We also assessed whether homolog families with CRCCs are enriched in certain protein secondary structural types. Initially, our analysis suggested that human proteins from homolog families with CRCCs showed a significant enrichment of domains with β-sheet secondary structure (**Fig 3C**). However, because proteins from families with CRCCs also have a longer average length (**S3A Fig**), we hypothesized that differences in protein length could be driving the observed differences in domain composition. Comparison of human proteins with CRCCs to a length-matched control set (similar sequence length but no CRCCs; see Methods), revealed that the difference in domain composition results from an association between domain composition and sequence length, rather than an association between the presence of CRCCs and secondary structure composition, including the presence of transmembrane helical domains (**S3B Fig**).

## Discussion

Synonymous mutations were once thought to be neutral. This assumption is the basis of the frequently used K_a_/K_s_ ratio, in which the synonymous substitution rate serves as a proxy for neutral mutational drift [*54,55*]. However, it is now widely accepted that codon usage in bacteria is shaped by selection [*56,57*]. The origins of eukaryotic (particularly mammalian) codon usage have remained more controversial [*58*], and it has been argued that the fitness effects of synonymous codon changes are too small to result in selection in species with a small population size [*59*]. However, more recent studies have shown that synonymous codon changes can have phenotypic effects [*20,60,61*] and that synonymous codon usage in eukaryotes is at least partly the result of selection [*58,62*].

Beyond synonymous codon selection in general, whether there are detectable patterns of codon usage within ORFs is still an open question. Previous studies have shown that the distribution of rare codons in ORFs is non-random in most organisms [*6*], however the functional significance has remained controversial, as have the evolutionary reasons (*i*.*e*., selection or drift). In recent years there has been a growing consensus that rare codons at the 5’ termini of coding sequences are conserved and increase translation efficiency [*13,15,16,43*]. In contrast, conservation of non-terminal rare codons is still under active debate, in part because of the many origins of codon usage bias, which can make it challenging to distinguish conservation of codon rarity versus other aspects of codon bias. For example, although a previous study identified low average codon usage frequencies within Pfam domain alignments [*36*], it was not determined whether these codons occurred more frequently than expected by random chance. Further, this study considered only absolute codon usage frequencies, which means that conservation of rare amino acids (e.g, cysteine), which are by definition encoded by codons that are rare in an absolute sense, can lead false-positive results. Likewise, Pechmann *et al*. analyzed rare codon conservation in several closely-related yeast species using a codon usage metric that compares tRNA supply with demand (as determined by mRNA levels) and found some evidence for conservation [*63*], although the relatively recent divergence of these species may make it more challenging to detect significant conservation.

To overcome the challenges of identifying conservation of codon rarity, we aligned synonymous codon usage frequencies across the ORFeomes of 76 diverse organisms and developed a null model capable of distinguishing conservation of codon rarity from other effects, including amino acid conservation, GC bias and codon pair bias. Our study demonstrates that clusters of rare codons are significantly conserved across much more distantly related species, spanning the tree of life, even after accounting for other known codon usage biases, including recently-diverged paralogs, %GC content and codon pair bias. The analysis framework described here can be used to analyze synonymous codon conservation in any organism with a fully sequenced genome. Our results suggest that synonymous codon usage is often subject to selection.

The conservation of rare codon clusters suggests they serve a functional role. Given the diverse effects of codon usage, it is likely that CRCCs have multiple functions. The hypothesis that codon usage modulates co-translational protein folding led to the expectation that the locations of rare codon clusters might be correlated with protein structural features. Studies examining correlations between codon usage and protein secondary structure have identified an enrichment of rare codons in unstructured regions and common codons in conserved, structured regions [*53,64*]. While rare codons in unstructured regions can also function to promote co-translational folding [*21,64*], their presence in such locations is also consistent with the hypothesis that rare codons exist due to mutational drift in genome regions under less selection for translational speed or accuracy [*53*]. The CRCCs identified by our study are not enriched at domain boundaries in human or *E*. *coli* coding sequences. If rare codons do function to separate the folding of protein structural units, these foldable units do not necessarily correspond to a defined domain. Our data set serves as a starting point for more detailed structural and functional analyses, including the effects of mRNA secondary structure on translation rate and co-translational folding of the encoded protein.

The association between CRCCs and the processes of growth and development is particularly intriguing, given that the coding sequences of cell cycle-regulated proteins are enriched in rare codons in general [*65*]. These results are consistent with results showing that substituting common codons for rare codons in the sequences encoding bacterial and fungal circadian regulatory proteins adversely affects the circadian clock and cell growth rate [*8,21*]. Of note, codon sensitivity in the fungal clock protein FRQ is localized to portions of the coding sequence encoding a predicted intrinsically disordered region (IDR) [*64*]. The functions of many IDRs include binding to other proteins and nucleic acids, often to regulate proliferation and cell cycle events [*66*]. Intriguingly, we found that CRCCs are enriched in proteins with binding functions (**Fig 2B**), suggest that synonymous codon usage may provide a mechanism to regulate cell growth and development across diverse species.

In conclusion, conservation of rare codons is a widespread phenomenon, and occurs in structurally and functionally diverse protein families. Homolog families with CRCCs were enriched in specific structural and functional categories. CRCCs were more likely to be found in water-soluble nuclear and cytosolic proteins rather than membrane proteins, suggesting a possible connection with domain organization and folding in the cytosol. Proteins with CRCCs are enriched in functions associated with development and cell growth. This association is particularly intriguing, given that codon usage has been shown to affect circadian growth rhythms of an organism [*8,21*], and both tRNA levels and the codon usage of highly expressed genes vary with cell growth rate and cell cycle stage [*65,67*]. The results reported here should be broadly useful toward the development of a mechanistic understanding of how synonymous codon usage can affect various aspects of protein biogenesis.

## Methods

### Selection of species for dataset

To minimize DNA identity within our dataset, we evaluated phylogenetic trees [*68*] constructed for species with fully sequenced genomes. A separate tree was constructed for species from each domain of life (eukaryotes, archaea, and bacteria) using 16*S* or 18*S* rRNA sequences aligned using MUSCLE [*42*]. 18*S* or 16*S* rRNA sequences were obtained from the Green Genes [*69*] or Silva rRNA [*70*] databases. Certain eukaryotic species (including *Giardia lamblia* and *Brugia malayi* in the final data set) were not present in the Silva database and their 18*S* rRNA sequences were obtained from NCBI. Based on these initial trees, closely related species were removed and the analysis repeated in order to maximize species diversity. Species for the final data set were chosen based primarily on diversity (to include representative species from the main branches of each tree) and secondarily on the significance of the organism (number of PubMed citations, etc.). Trees were drawn using Plottree [*68*] for an unrooted tree. The final 76 species used for rare codon conservation analysis are listed in **S1–S3 Tables**, and include 24 bacteria, 26 archaea, and 26 eukaryotes.

### ORFeome data

For each of the 76 selected species, the set of all annotated protein coding sequences in the fully sequenced genome (the ORFeome) was collected. Most ORFeomes were obtained by downloading the coding sequences corresponding to all protein coding genes in the species genome from the NCBI database. To avoid fragments not corresponding to full reading frames, only those gene sequences with length equal to an integer multiple of 3 were included in the final ORFeomes. If the same gene identifier was associated with >1 sequence (for example, multiple splice isoforms for some eukaryotic sequences), only the longest sequence was used. *Ixodes scapularis* cDNA was not available from NCBI, so the transcript set was downloaded from Vector Base [*71*].

### Assignment and alignment of homolog families

Families of homologous genes from the 76 ORFeomes were assembled using OrthoMCL [*41*]. Families were edited to remove potential false positives arising from paralogs by including a maximum of one protein sequence from each species. The representative sequence was chosen at random and other sequences from the same species were discarded. For each resulting homolog family, protein sequences were aligned using MUSCLE [*42*].

### Locations of rare codon clusters

Overall codon usage for each species was determined by counting occurrences of each codon in the corresponding ORFeome. To calculate the relative codon usage along each gene, we used the %MinMax algorithm [*6*], which was designed to identify clusters of synonymous rare codons. %MinMax compares actual codon usage to hypothetical sequences encoding the same amino acids using either the most common (%MinMax = +100) or most rare (%MinMax = -100) synonymous codons for the species of origin. To identify clusters of rare codons, %MinMax scores were averaged over a sliding window of 17 codons, and one or more consecutive windows with %MinMax < 0 were considered a rare codon cluster. For each cluster, the “peak” was defined as the window with the minimum (most negative) %MinMax score.

### Identifying statistically significant rare codon co-occurrence

To pinpoint specific rare codon clusters that co-occur, we used the following statistical test. As for the broad test across the entire dataset (see Results and Supplementary Methods), rare codon clusters in two homolog sequences were considered to co-occur if their peaks fell within a distance of +/- 2 positions in a homolog family. For an alignment column with aligned peaks in *m* out of *n* homologs (*m*≥1), the p-value of co-occurrence = P(X≥*m*|X≥1). X follows a binomial distribution (*n*, *p*0) where *p*0 is the probability of a position being a peak (determined by the total number of peaks and the length of the alignment).

### Trimming sequence termini

To separate rare codon conservation within coding sequences versus rare codon co-occurrence at sequence termini [*16,43*], we located the most N-terminal position where no gaps were found in a homolog family alignment, and discarded any p-values within the next 50 codons. The same process was repeated for the 50 codons preceding the most C-terminal gap in the alignment. This filtering considered gaps because trimming the first 50 codons in the alignment without considering gaps did not trim all N-terminal regions where co-translational folding effects are expected to be minimal (i.e., before 20 aa of the nascent chain has emerged from the ribosome exit tunnel). This method proved appropriate for homolog families of similar sized proteins while not over or under penalizing diverse families containing homologs from diverse species spanning the tree of life. In subsequent analyses, homolog families were only used if a position within the trimmed homolog family alignment had a p-value below the specific threshold considered.

### Random reverse translations (RRTs)

To construct RRTs based on %GC-specific codon usage data, all coding sequences in an ORFeome were sorted into partially overlapping GC3 content bins (for example, sequences with %GC3 of 20–30%, 25–35%, 30–40%, etc.). Overlapping bins were used to increase the number of sequences per bin for more accurate codon usage data while still keeping the average %GC3 content for adjacent bins close together, so that every sequence will have a %GC3 close to the average %GC3 of a sequence bin. Binning was based on %GC3 rather than overall %GC because %GC is correlated with amino acid content and binning by %GC3 is therefore more effective for replicating the %GC content of the original sequence. The codon frequencies within each sequence bin were counted, and the corresponding %GC-biased codon frequency table was used to construct the RRT sequences for each coding sequence.

In addition, certain codon pairs are over- or under-represented in some ORFeomes, and this bias was accounted for in the RRT as a codon pair multiplier. A codon pair multiplier indicates the enrichment (or under-enrichment) of codon A at the -1 position with respect to codon B (5’-A-B-3’), relative to the average local usage frequency of A codons near B codons. For example, to calculate the codon pair multiplier for encoding Leu with CTA before TGC, the average usage frequency of CTA is calculated for all Leu residues within 17 codon windows centered on all TGC codons, with the exception of Leu at the -1 position. The usage frequency of CTA at the -1 position is calculated separately, and the ratio of these two usage frequencies (CTA frequency at -1/average local CTA frequency) gives the codon pair multiplier.

RRTs were constructed from 3’ to 5’. At each position, a codon encoding the appropriate amino acid was selected randomly but biased by the count of a codon in a %GC3 bin and multiplied by the codon pair multiplier for each following codon, in order to obtain a position-specific codon usage number. In this way, the enrichment or under-enrichment of a specific codon is relative to both the local %GC content of the coding sequence and the codon pair multiplier, which indicates how much the usage of a codon should be enriched or under-enriched based on the identity of the following 3’ codon (which is chosen first during the RRT).

For each gene in each homolog family, 200%GC-matched, codon pair-matched RRTs were constructed. Each set of RRTs was analyzed for co-occurrence of rare codons to determine which sites of significant co-occurrence were likely caused by amino acid bias. For each position in a homolog family alignment, we determined how many RRTs had significant co-occurrence at that position. For all alignment positions that had significant co-occurrence in ≥ 1 RRT, we identified the 5% of positions with the highest number of RRTs with significant (p-value < 1x10^-4^) co-occurrence, as these positions are likely to be subject to sequence bias and were therefore regarded as suspect. We disregarded any positions within +/- 8 codons of a suspect alignment position, as they fall within the same %MinMax window.

### Gene ontology enrichment analysis

Blast2Go [*72*] was used to assign gene ontology (GO) terms to each protein in a homolog family. For each GO term we counted the number of homolog families with significant rare codon conservation that have an ORF assigned with this GO term, and all other instances of that GO term. We then create a contingency table with these counts for each GO term and performed an enrichment analysis using Fisher’s exact test. GO terms were also divided by class (cellular composition, molecular function, and biological process) and the 10 most common terms from each class were analyzed for enrichment.

### CATH and SCOP domains from PDB

A PDB BLAST database was constructed from all non-identical protein chains in the PDB by selecting the PDB representative structure, which selects the highest quality structure if more than one structure is available for identical protein sequences. Proteins from the 76 ORFeome dataset were assigned PDB matches based on BLASTP. A match was required to contain an alignment with ≥95% sequence identity. SCOP and CATH domain annotations were downloaded from the PDB. 8.4% of homolog families had a PDB CATH assignment, and 7.8% had a PDB SCOP assignment.

### Domain boundary assignments for human and E. coli genomes

Domain assignments for the human and *E*. *coli* genomes were downloaded from the Gene3D database (ftp://ftp.biochem.ucl.ac.uk/pub/gene3d_data/CURRENT_RELEASE/). These domains were assigned to human and *E*. *coli* coding sequences in the conservation data set by matching gene names.

### Locations of CRCCs relative to protein domains

Global and local enrichment of significant CRCCs (p < 0.05; post RRT correction) relative to domain boundaries was assayed using CATH, SCOP and Ensembl domains and multiple binomial tests. To test for overall enrichment, we first considered any CRCC within 10 codons of a domain boundary to be “near” such boundaries; all others to be “far.” Using the size of the proteins and domains without gaps, we next determined if more (or fewer) CRCCs occurred near boundaries than expected when compared to a null model where CRCCs were distributed evenly across alignments. The same method was used to determine whether more or fewer CRCCs occurred within domains than expected.

Finally, we assessed fixed 50 residue windows near N-terminal boundaries versus C-terminal boundaries for windows that: (1) followed domain boundaries; (2) were centered on domain boundaries; or (3) flanked domain boundaries. Since the windows were equal size, we first accessed if more (or fewer) CRCCs were found near N terminal boundaries as compared to C terminal boundaries for each protein using a binomial test with p = 0.5. We also considered N versus C terminal boundaries combined across all predicted domains, and compared results with or without the N-terminal trimming procedure described above.

### Sequence length control set

Sequences with conserved rare codon clusters (CRCCs) have longer average length than sequences without CRCCs. To control for differences in domain composition related to differences in length, a length-matched control set was assembled. Each human protein in a homolog family with CRCCs (p-value ≤ 1E-4) was matched to the human protein with the most similar length (minimum absolute value of length difference) from a homolog family without CRCCs. Each protein was only included once in the control set.

## Supporting information

S1 Text Calculation of the p-value for co-occurrence of rare codon clusters. (PDF)

S1 Table Bacterial species used in this study. (PDF)

S2 Table Archaeal species used in this study. (PDF)

S3 Table Eukaryotic species used in this study. (PDF)

S1 Fig Phylogenetic tree of 76 species used in co-occurrence analysis. The tree is constructed from 16*S* and 18*S* rRNA sequences. See **S1–S3 Tables** for the names of all species used.
(PDF)

S2 Fig %MinMax and tAI are both accurate predictors of relative translation rate. (**A**) Synonymous mutations made in an 18-codon window near the 5’ end of the coding sequence incoding the C-terminal half domain of the translation rate biosensor YKB [*23*] predictably altered translation rate. Rare synonymous mutations (lower %MinMax values) led to an increase the [YK]/[KB] molar ratio, indicating slower translation rates. (**B**) The geometric mean of tAI values for the same mutations in (A) similarly predicted slower translation rates.
(PNG)

S3 Fig **A.** Average length of human and *E*. *coli* proteins in homolog families with or without CRCCs. **B.** Length differences do not explain lower percentage of membrane proteins or higher frequency of rare codons (larger average %%Min) in sequences from homolog families with CRCCs. Graphs compare the full set of analyzed human sequences, human sequences from homolog families with CRCCs (p-value ≤ 1E-4), and a length-matched control set (similar lengths to CRCCs set but no CRCCs). %TMH = percentage of proteins with ≥ 1 transmembrane helix predicted by TMHMM. Average %%Min = average percent of sequence windows containing RCCs (%MinMax < 0).
(TIF)

S4 Fig An example of the CRCC analysis output for alpha-D-glucose-1-P phosphatase. Green bars in the heatmap indicates the location of rare codons and p-values for co-occurrence of rare codons in the sequence alignment (minimum p-value = 5E-6). The structure of the *E*. *coli* homolog is shown (PDBID 2B0C), color-coded as for **Fig 4**, to indicate portions of the protein outside the ribosome exit tunnel and able to fold at two rare codon-induced translational pauses. Locations of these CRCCs are indicated on the alignment by arrows. The contact maps indicate amino acids pairs that are in contact (distance ≤ 6 Å) in the three-dimensional structure.
(TIF)
