# Supplementary material for: Widespread position-specific conservation of synonymous rare codons within coding sequences
Source: PLoS Comput Biol. 2017 May 5;13(5):e1005531. doi: 10.1371/journal.pcbi.1005531 (PMC5438181; doi:10.1371/journal.pcbi.1005531)
Supplement: S1 Text — (PDF) [file pcbi.1005531.s001.pdf]

Supplementary Text for:

Widespread Position-specific Conservation of Synonymous Rare Codons Within Coding Sequences

Julie L. Chaney[1#a], Aaron Steele[2], Rory Carmichael[2#b], Alicia T. Specht[3], Kim Ngo[1,2], Jun Li[3], Scott Emrich[2]* & Patricia L. Clark[1,4]*

[1]*Department of Chemistry & Biochemistry,* [2]*Department of Computer Science & Engineering,* [3]*Department of Applied and Computational Mathematics & Statistics, and* [4]*Department of Chemical & Biomolecular Engineering,*

University of Notre Dame, Notre Dame, Indiana, United States of America

Short Title: Widespread Position-specific Conservation of Synonymous Codon Rarity

**Corresponding authors:*

email: pclark1@nd.edu (PLC); semrich@nd.edu (SE)

[#a]*Current address:*

Siemens Healthineers, 3400 Middlebury Street, Elkhart, Indiana, United States of America

[#b]*Current address:*

Quantcast, 201 3rd Street, San Francisco, California, United States of America

Supplementary Methods

Calculation of the p-value for co-occurrence of rare codon clusters

The hypothesis tested in this section is whether rare codon clusters, as a whole, are conserved across homologs. This test was not designed to identify *which* rare codon clusters are conserved, which was done using the binomial test described in the main text. Instead, this test counted the total number of co-occurring rare codon clusters observed in the homolog families, and compared this number with the total number of co-occurring clusters under the null hypothesis to get a p-value. The total number of co-occurring clusters was generated using a sliding window strategy.

We first pre-processed the data set to remove gaps in the alignment, as these will complicate the sliding window approach. We deleted all alignment positions that have gaps in one or more of the homologs. Then we discarded all homolog families with alignments <10 amino acids long. Alignments this short have little power in testing the co-occurrence hypothesis. Because of this, although the threshold of 10 amino acids is arbitrary we expect the results to be insensitive to this threshold. After this pre-processing, 15764 out of 16520 homolog families were retained for further analysis. The above pre-processing does not introduce bias to our analysis, and since more than 95% of the homolog families remain in our data set, the loss of power should not be significant.

To test whether rare codons as a whole co-occur across this dataset, we performed the following statistical test. For homolog family g, suppose its alignment length is L_g, and the number of homologs in this family is S_g. Let $X_{gij} = 1$ if position i in

homolog j of homolog family g is a rare codon cluster center, and 0 otherwise. Then the number of overlapping cluster centers in homolog j_1 and homolog j_2 is

$$\sum_{i=1}^{L_g} X_{gij_1} X_{gij_2}.$$

So the average number of overlapping centers in all homologs is

$$C_g = \frac{1}{S_g(S_g - 1)} \sum_{j_1, j_2 = 1, \cdots, S_g, j_1 \neq j_2} \sum \sum_{i=1}^{L_g} X_{gij_1} X_{gij_2}.$$

To determine a p-value, we estimate the distribution of C_g under the null distribution by permuting the locations of rare codons. Since the rare codons are clustered, we designed a permutation to preserve the spatial relations between codons by sliding the homolog sequences relative to each other and calculating the average number of overlapping centers. That is, we define

$$X_{gij}^{(*k)} = \begin{cases} X_{gi(j-k)} & \text{if } j > k, \\ X_{gi(j-k+L_g)} & \text{otherwise.} \end{cases}$$

$X_{gij}^{(*k)}$ is the new homolog j that has been slid k amino acids to the right. Then, we calculate

$$C_g^{(*k)} = \frac{1}{S_g(S_g - 1)} \sum_{j_1, j_2 = 1, \cdots, S_g, j_1 \neq j_2} \sum \sum_{i=1}^{L_g} X_{gij_1}^{(*k)} X_{gij_2}.$$

$C_g^{(*0)}, \ldots, C_g^{(*L_g)}$ comprise the permutation-based null distribution of C_g.

Further, we define

$$\mu_g = \frac{1}{L_g} \sum_{k=1}^{L_g} C_g^{(*k)},$$

$$\sigma_g^2 = \frac{1}{L_g - 1} \sum_{k=1}^{L_g} (C_g^{(*k)} - \mu_g)^2.$$

μ_g and σ_g^2 are the estimated mean and variance of the null distribution of C_g. A z statistic can be defined by

$$z_g = \frac{C_g - \mu_g}{\sqrt{\sigma_g^2}}.$$

z_g may roughly follow standard normal distribution, but the approximation may be very inaccurate. Although using z_g to test the significance of each homolog family represents an approximation, together they can accurately test the significance of the co-occurrence of all groups. Let

$$z = \frac{\sum_{g=1}^{G} C_g - \sum_{g=1}^{G} \mu_g}{\sqrt{\sum_{g=1}^{G} \sigma_g^2}},$$

where G is the total number of families. Central limit theorem guarantees that z follows a standard normal distribution.

Finally, we get $z = 66.5$, corresponding to $p\text{-value} < 1 \times 10^{-300}$.
