# Supplementary material for: Widespread position-specific conservation of synonymous rare codons within coding sequences
Source: PLoS Comput Biol. 2017 May 5;13(5):e1005531. doi: 10.1371/journal.pcbi.1005531 (PMC5438181; doi:10.1371/journal.pcbi.1005531)
Supplement: S1 Table — (PDF) [file pcbi.1005531.s002.pdf]

ID	species
Aaeo	*Aquifex aeolicus* VF5
Bant	*Bacillus anthracis* str. Ames
Bbur	*Borrelia burgdorferi* ZS7
Bfra	*Bacteroides fragilis* YCH46
Bmel	*Brucella melitensis* bv. 1 str. 16M
Bper	*Bordetella pertussis* Tohama I
Cbur	*Coxiella burnetii* RSA 493
Cjej	*Campylobacter jejuni* subsp. *jejuni* 81-176
Ctep	*Chlorobium tepidum* TLS
Dalk	*Desulfurivibrio alkaliphilus* AHT2
Drad	*Deinococcus radiodurans* R1
Ecol	*Escherichia coli* str. K-12 substr. MG1655
Hpyl	*Helicobacter pylori* 26695
Mtub	*Mycobacterium tuberculosis* CDC1551
Nmen	*Neisseria meningitidis* MC58
Nsp	*Nostoc sp. PCC 7120*
Pflu	*Pseudomonas protegens* Pf-5
Rmet	*Cupriavidus metallidurans* CH34
Saur	*Staphylococcus aureus* subsp. *aureu*s N315
Spne	*Streptococcus pneumonia*e CGSP14
Tmar	*Thermotoga maritima* MSB8
Tthe	*Thermus thermophilus* HB27
Xfas	*Xylella fastidiosa* Temecula1
Ypes	*Yersinia pestis* KIM10+

Table S1. Bacterial species used in this study.
