# Supplementary material for: Widespread position-specific conservation of synonymous rare codons within coding sequences
Source: PLoS Comput Biol. 2017 May 5;13(5):e1005531. doi: 10.1371/journal.pcbi.1005531 (PMC5438181; doi:10.1371/journal.pcbi.1005531)
Supplement: S2 Table — (PDF) [file pcbi.1005531.s003.pdf]

ID	species
Aboo	*Aciduliprofundum boonei* T469
Aful	*Archaeoglobus fulgidus* DSM 4304
Asac	*Acidilobus saccharovorans* 345-15
CKor	*Candidatus Korarchaeum* cryptofilum OPF8
Dkam	*Desulfurococcus kamchatkensis* 1221n
Hbut	*Hyperthermus butylicus* DSM 5456
Hjeo	*Halalkalicoccus jeotgali* B3
Hsal	*Halobacterium salinarum* R1
Hvol	*Haloferax volcanii* DS2
Meve	*Methanohalobium evestigatum* Z-7303
Mfer	*Methanothermus fervidus* DSM 2088
Mhun	*Methanospirillum hungatei* JF-1
Mkan	*Methanopyrus kandleri* AV19
Mlab	*Methanocorpusculum labreanum* Z
Mmar	*Methanococcus maripaludis* S2
Msed	*Metallosphaera sedula* DSM 5348
Msta	*Methanosphaera stadtmanae* DSM 3091
Mthe	*Methanosaeta thermophila* PT
Nequ	*Nanoarchaeum equitans* Kin4-M
Nmar	*Nitrosopumilus maritimus* SCM1
Pars	*Pyrobaculum arsenaticum* DSM 13514
Ptor	*Picrophilus torridus* DSM 9790
Ssol	*Sulfolobus solfataricus* P2
Taci	*Thermoplasma acidophilum* DSM 1728
Tbar	*Thermococcus barophilus* MP
Tpen	*Thermofilum pendens* Hrk 5

Table S2. Archaeal species used in this study.
