# Supplementary material for: Widespread position-specific conservation of synonymous rare codons within coding sequences
Source: PLoS Comput Biol. 2017 May 5;13(5):e1005531. doi: 10.1371/journal.pcbi.1005531 (PMC5438181; doi:10.1371/journal.pcbi.1005531)
Supplement: S3 Table — (PDF) [file pcbi.1005531.s004.pdf]

| ID | species | common name |
|---|---|---|
| Afum | *Aspergillus fumigatus* Af293 | |
| Agam | *Anopheles gambiae* | mosquito |
| Amel | *Apis mellifera* strain DH4 | bee |
| Atha | *Arabidopsis thaliana* | |
| Bmal | *Brugia malayi* | |
| Cele | *Caenorhabditis elegans* | worm |
| Cneo | *Cryptococcus neoformans* var. *neoformans* JEC21 | |
| Crei | *Chlamydomonas reinhardtii* | |
| Ddis | *Dictyostelium discoideum* AX4 | |
| Dmel | *Drosophila melanogaster* | fly |
| Drer | *Danio rerio* | zebrafish |
| Ecun | *Encephalitozoon cuniculi* GB-M1 | |
| Ehis | *Entamoeba histolytica* HM-1:IMSS | |
| Ggal | *Gallus gallus* | chicken |
| Glam | *Giardia lamblia* ATCC 50803 | |
| Hsap | *Homo sapiens* | human |
| Isca | *Ixodes scapularis* | tick |
| Lmaj | *Leishmania major* strain Friedlin | |
| Mmus | *Mus musculus* | mouse |
| Oana | *Ornithorhynchus anatinus* | platyus |
| Pfal | *Plasmodium falciparum* 3D7 | |
| Scer | *Saccharomyces cerevisiae* S288c | yeast |
| Spur | *Strongylocentrotus purpuratus* | urchin |
| Tbru | *Trypanosoma brucei gambiense* DAL972 | |
| Tpar | *Theileria parva* strain Muguga | |
| Xtro | *Xenopus (Silurana) tropicalis* | frog |

**Table S3.** Eukaryotic species used in this study.
