# Supplementary figures and images for: Widespread position-specific conservation of synonymous rare codons within coding sequences
Source: PLoS Comput Biol. 2017 May 5;13(5):e1005531. doi: 10.1371/journal.pcbi.1005531 (PMC5438181; doi:10.1371/journal.pcbi.1005531)

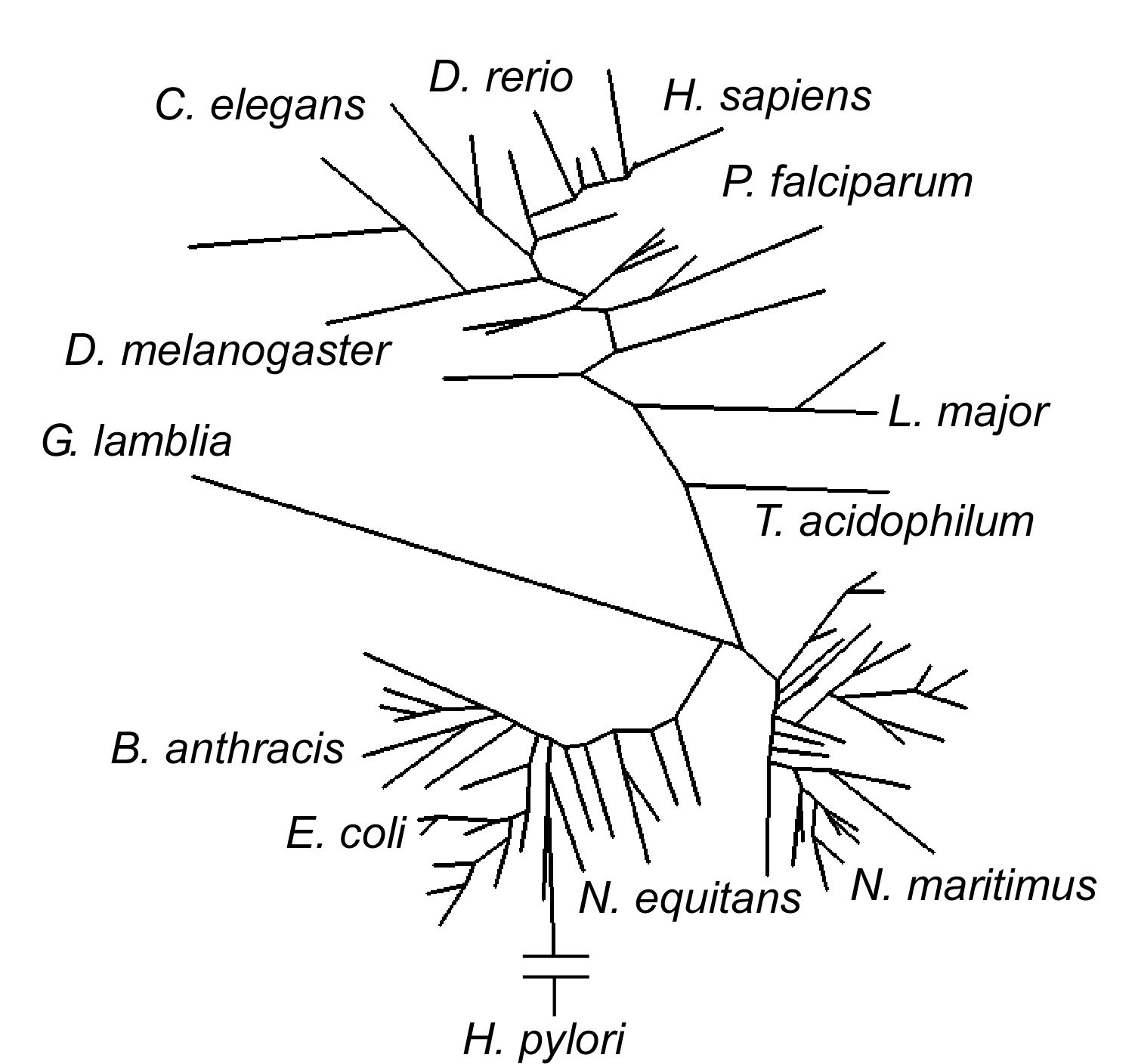

Supplement: S1 Fig — The tree is constructed from 16S and 18S rRNA sequences. See S1–S3 Tables for the names of all species used. (PDF) [file pcbi.1005531.s005.pdf]

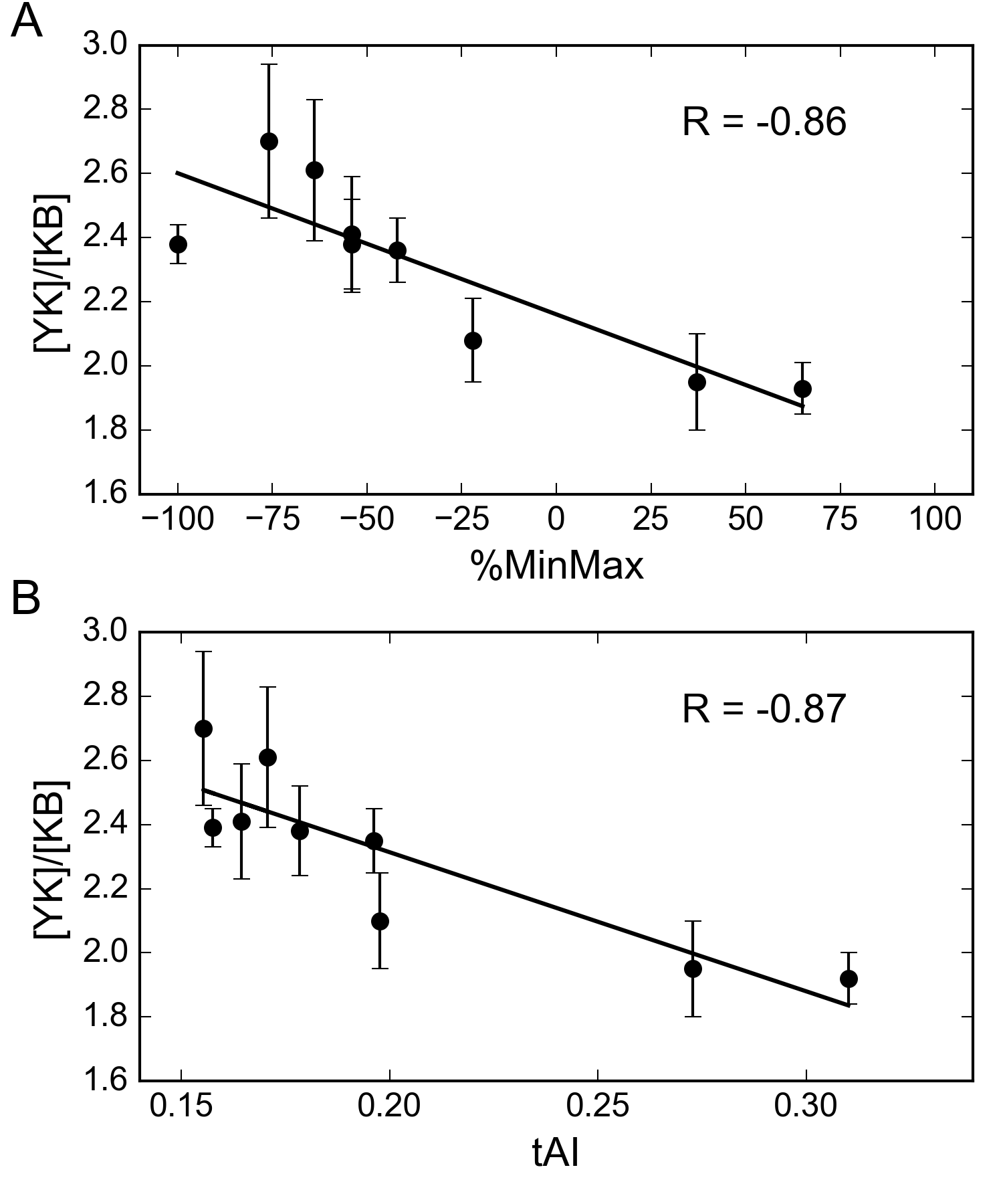

Supplement: S2 Fig — (A) Synonymous mutations made in an 18-codon window near the 5’ end of the coding sequence incoding the C-terminal half domain of the translation rate biosensor YKB [23] predictably altered translation rate. Rare synonymous mutations (lower %MinMax values) led to an increase the [YK]/[KB] molar ratio, indicating slower translation rates. (B) The geometric mean of tAI values for the same mutations in (A) similarly predicted slower translation rates. (PNG) [file pcbi.1005531.s006.png]

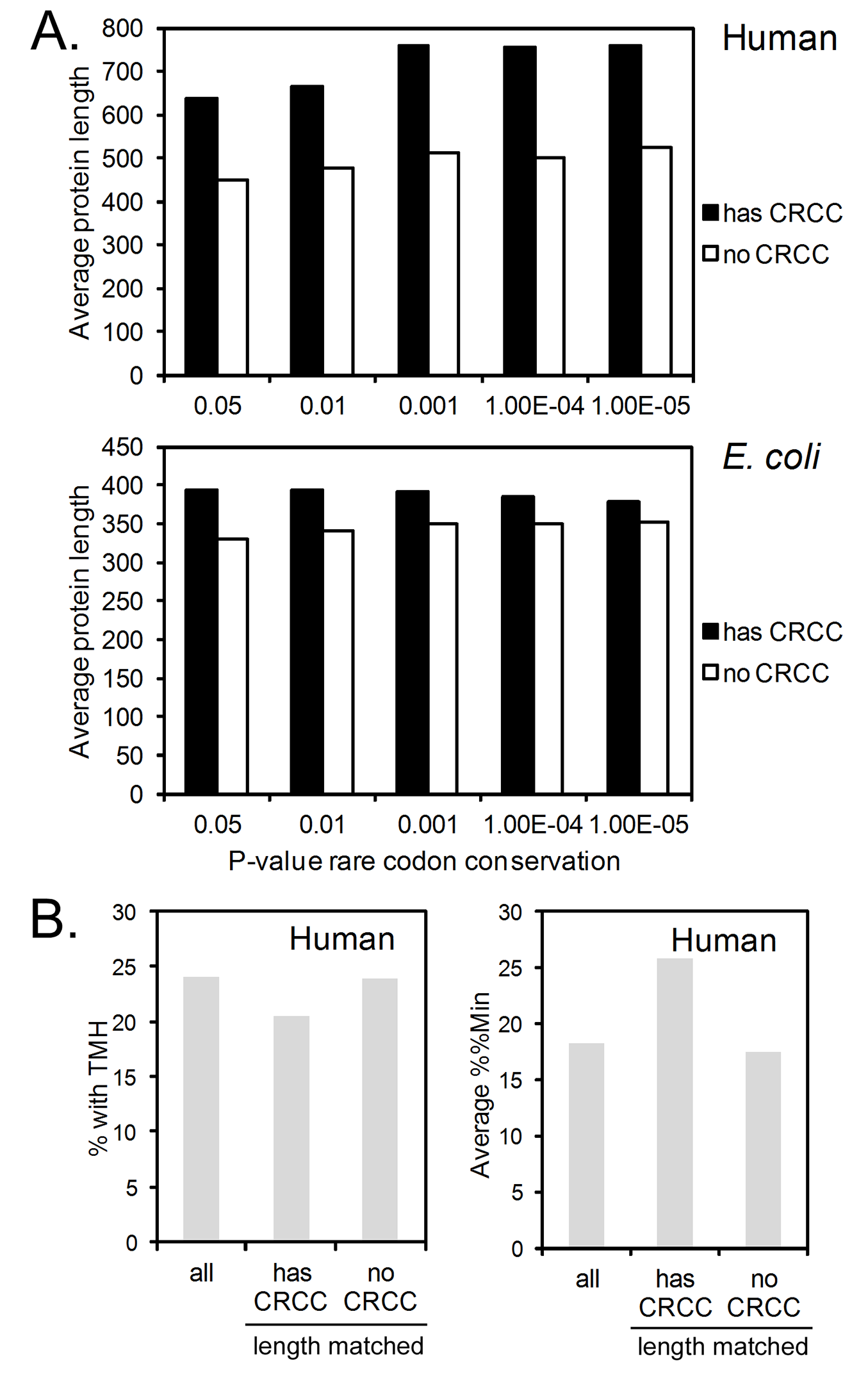

Supplement: S3 Fig — A. Average length of human and E. coli proteins in homolog families with or without CRCCs. B. Length differences do not explain lower percentage of membrane proteins or higher frequency of rare codons (larger average %%Min) in sequences from homolog families with CRCCs. Graphs compare the full set of analyzed human sequences, human sequences from homolog families with CRCCs (p-value ≤ 1E-4), and a length-matched control set (similar lengths to CRCCs set but no CRCCs). %TMH = percentage of proteins with ≥ 1 transmembrane helix predicted by TMHMM. Average %%Min = average percent of sequence windows containing RCCs (%MinMax < 0). (TIF) [file pcbi.1005531.s007.tif]

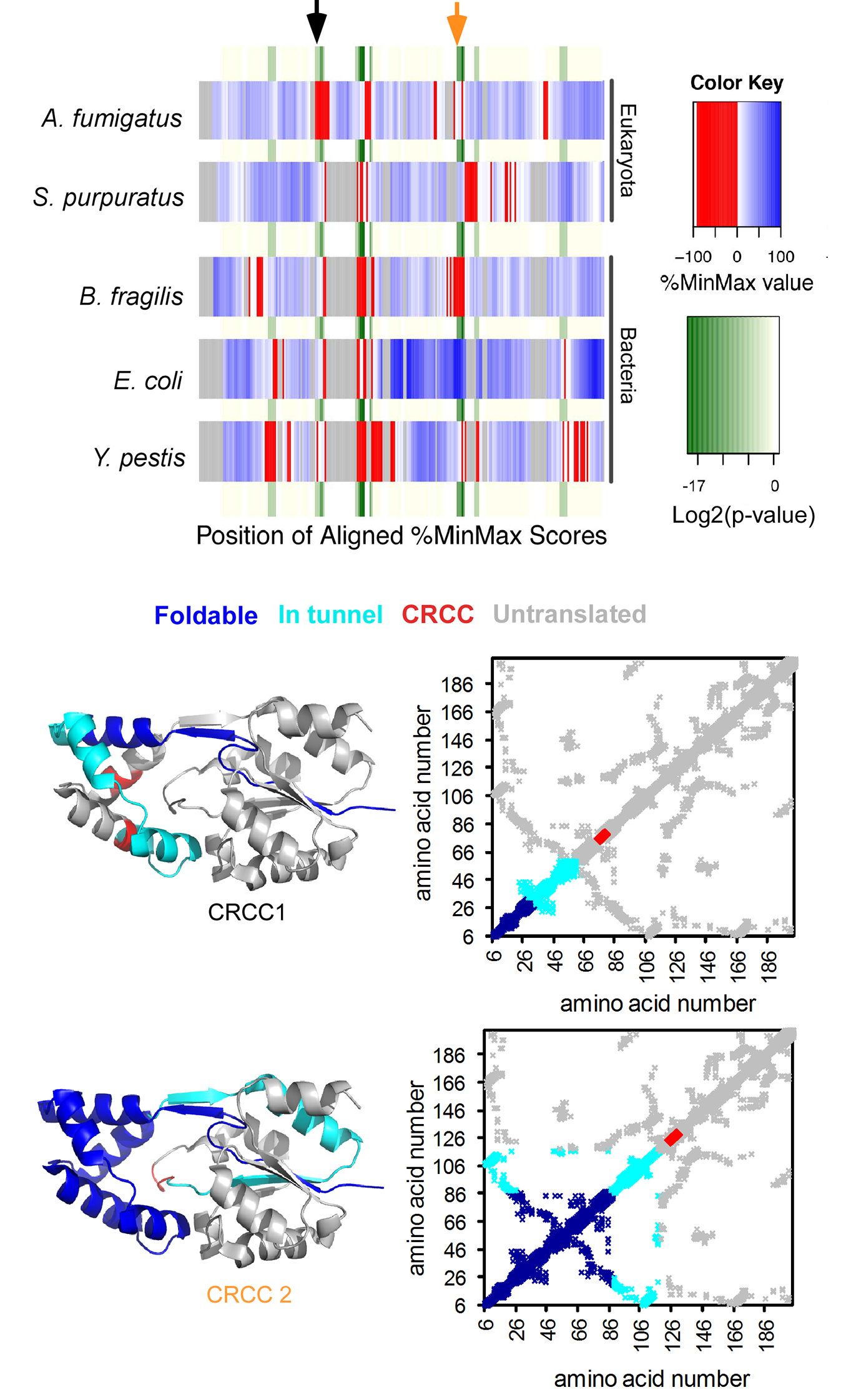

Supplement: S4 Fig — Green bars in the heatmap indicates the location of rare codons and p-values for co-occurrence of rare codons in the sequence alignment (minimum p-value = 5E-6). The structure of the E. coli homolog is shown (PDBID 2B0C), color-coded as for Fig 4, to indicate portions of the protein outside the ribosome exit tunnel and able to fold at two rare codon-induced translational pauses. Locations of these CRCCs are indicated on the alignment by arrows. The contact maps indicate amino acids pairs that are in contact (distance ≤ 6 Å) in the three-dimensional structure. (TIF) [file pcbi.1005531.s008.tif]
